# An open-label, multicenter study to evaluate the safe and effective use of the single-use autoinjector with an Avonex^® ^prefilled syringe in multiple sclerosis subjects

**DOI:** 10.1186/1471-2377-11-126

**Published:** 2011-10-14

**Authors:** J Theodore Phillips, Edward Fox, William Grainger, Dianne Tuccillo, Shifang Liu, Aaron Deykin

**Affiliations:** 1 Texas Neurology, 6301 Gaston Ave, West Tower, #100, Dallas, Texas, USA; 2 MS Clinic of Central Texas, Central Texas Neurology Consultants, PA, 16040 Park Valley Drive, Building B, Suite 100, Round Rock, Texas, USA; 3 Neurological Physicians of Arizona, Clinical Research Advantage, 726 N. Greenfield Road, Suite 110, Gilbert, Arizona, USA; 4 Biogen Idec Inc., 14 Cambridge Center, Cambridge, Massachusetts, USA

## Abstract

**Background:**

The ability to self-inject in patients with multiple sclerosis (MS) has been associated with a reduced risk of missed injections and drug discontinuation, and a beneficial effect on patients' independence. However, injection anxiety, needle phobia and disease-related disability are major barriers to a patient's ability to self-administer treatment. Use of an autoinjector may improve patients' ability to self-inject. This study evaluated the safe and effective use of Avonex Pen™ (prefilled pen), a single use autoinjector, for intramuscular delivery of interferon beta-1a (IM IFNβ-1a, Avonex) in MS patients.

**Methods:**

This was a Phase IIIb, open-label, single-country, multicenter trial in MS patients currently using IM IFNβ-1a prefilled syringes. Patients received weekly 30 mcg IM IFNβ-1a treatment over 4 weeks. On Day 1, patients self-administered IM IFNβ-1a using a prefilled syringe at the clinic. On Day 8, patients received training on the prefilled pen and self-administered IM IFNβ-1a using the device. On Day 15, patients self-administered IM IFNβ-1a at home using the prefilled pen. A final injection occurred at the clinic on Day 22 when patients self-administered IM IFNβ-1a using the prefilled pen while clinic staff observed and completed a detailed questionnaire documenting patients' ability to self-inject with the device. Serum neopterin levels were evaluated pre and post-injection on Days 1 and 8. Adverse events were monitored throughout.

**Results:**

Seventy-one (96%) patients completed the study. The overall success rate in safely and effectively using the prefilled pen was 89%. No device malfunctions occurred. One unsuccessful administration occurred at Day 22 due to patient error; no patient injury resulted. Patients gave the prefilled pen high ratings (8.7-9.3) on a 10-point scale for ease of use (0 = extremely difficult, 10 = extremely easy). Ninety-four percent of patients preferred the prefilled pen over the prefilled syringe. Induction of serum neopterin levels, serving as a biomarker for type 1 interferon action, was similar to that of the prefilled syringe. The prefilled pen demonstrated a safety profile comparable to the prefilled syringe.

**Conclusions:**

The prefilled pen is a safe and effective device for administration of IM IFNβ-1a and represents an alternative method for self-injection for MS patients using this therapy.

**Trial registration:**

This study is registered at clinicaltrials.gov, identifier: NCT00828204

## Background

Multiple sclerosis (MS) is a chronic inflammatory disease of the central nervous system that can lead to extensive neurodegeneration and subsequent irreversible disability. Symptoms of MS affect motor, sensory, visual, and autonomic systems but are not limited to these areas alone [1]. While there is no cure for MS, treatment with disease modifying therapies (DMTs) can reduce the frequency of relapses and disability associated with the disease [2-5].

For patients prescribed injectable DMTs, self-administration has been associated with a reduced risk of missed injections and drug discontinuation, and a beneficial effect on patients' sense of independence [6-8]. However, patients with MS face a variety of challenges that limit their ability to self-administer treatment [9]. Injection anxiety, including anticipation of pain, fear of hitting bone, inability to complete the injection effectively and fear of needle breakage, is an important barrier to self-injection [10]. Needle phobia, a component of injection anxiety, occurs in approximately 50% of patients with MS and therefore presents a significant concern for patients using injectable therapies [11]. Furthermore, as MS is a disease that affects the CNS, additional challenges to self-injection develop as the disease progresses and disability accumulates. Impairment of fine motor skills and decreased coordination present obstacles to the independent use of self-injected MS therapies [12].

The clinical importance of enabling self-injection is supported by various studies that have shown that the injection process can be made easier for patients through the use of automated injectors [13-16]. Autoinjectors have been shown to improve treatment adherence, reduce injection related adverse events (AEs) such as pain, and decrease injection anxiety [17-19]. Given these clinical benefits and recognizing the frequency at which barriers to self-injection are encountered by MS patients, including those using intramuscular interferon beta-1a (IM IFNβ-1a, Avonex) [11,20], it is evident that a mechanism to facilitate a patient's ability to self-inject IM IFNβ-1a is needed.

The Avonex Pen™ (prefilled pen) is a single use autoinjector containing the commercially available Avonex^® ^Prefilled Syringe for once-weekly intramuscular (IM) injection. The prefilled pen has been developed as the first IM autoinjector available for long-term treatment with IM IFNβ-1a. Features of the prefilled pen have been specifically designed to overcome the barriers to self-injection and facilitate the technical aspects of the injection process. These features include a protective sheath that conceals the needle within the device prior to injection, automated needle insertion and medication dispensing, a diameter and length designed to stabilize the device during the injection process, a safety mechanism which prevents accidental injection, and a visual indicator to confirm the full dose has been administered.

The prefilled pen has the potential to improve injection methods and simplify the self-injection process, thereby addressing an unmet need in patients using IM IFNβ-1a. This study was conducted to evaluate the safe and effective use of the prefilled pen for IM delivery of IFNβ-1a in patients with MS.

## Methods

### Study Design

This was a Phase IIIb, open-label, single-arm, multicenter study to evaluate the safe and effective use of the prefilled pen. A total of 17 sites in the United States participated. Investigators at each site obtained institutional review board (IRB) approval for the study protocol. This study was performed in accordance with all international, federal and local regulations, and written informed consent was obtained from each patient prior to eligibility evaluations. Study duration was 6 weeks, including a 14-day screening period and a 4 week IM IFNβ-1a treatment period. There were a total of 9 scheduled clinic visits per patient.

### Study Population

Study participants were 18 to 65 years of age. Eligibility criteria required patients to have been self-administering the prefilled syringes to treat MS for the 12 weeks prior to the screening visit. Key exclusion criteria included concomitant treatment with prescribed immunomodulators or immunosuppressants, and unwillingness or inability to comply with the requirements of the protocol, including the presence of any condition (physical, mental, or social) that was likely to affect a patient's ability to return for follow up visits on schedule.

### Device Description

**Figure 1:**
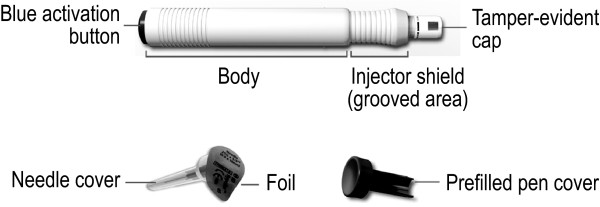
**Avonex Pen**.

The prefilled pen is shown in Figure 1. This device uses two springs to deliver the IM IFNβ-1a dose. The first spring performs the needle insertion and the second spring dispenses the medication. Activation of the second spring is dependent upon the successful completion of the first spring's activity. The prefilled pen is 13.5 cm in length and has a diameter of 1.5 cm. A 25 G × 5/8 inch (16 mm) needle, housed in a protective shield within the device, is used to deliver the IM IFNβ-1a dose.

For the manual injection with the prefilled syringe (Injection 1), patients used their own supply of IM IFNβ-1a prefilled syringes. The needle size used for Injection 1 was not recorded for each patient since the needle size used for manual injection of the prefilled syringe can vary between patients. A 23 G × 1.25 inch (32 mm) needle is included in the commercially available IM IFNβ-1a prefilled syringe package, however a different needle size (25 G × 1 inch) is also approved for use with the IM IFNβ-1a prefilled syringe. The decision of which needle a patient should use is based on the needs of the patient and left to the discretion of the prescribing physician, per IM IFNβ-1a prescribing information.

### Treatment

All patients received 30 mcg doses of IM IFNβ-1a once weekly over 4 weeks. Patients who were taking prophylactic therapy for flu-like symptoms at the start of the study continued the same medication and dose until their participation in the study was completed. If MS relapses occurred during the study, these were treated at the Investigator's discretion following standard medical practice, as long as treatment did not involve any of the protocol excluded concomitant medications. Treatment for spasticity, fatigue, or other MS associated symptoms was not restricted.

### Treatment Schedule

Patients who met the eligibility criteria during the 14-day screening period entered into a 4-week IM IFNβ-1a treatment period. Injection 1 took place on Day 1 and was administered at the study clinic by the patient using a prefilled syringe from their own supply of IM IFNβ-1a prefilled syringes and needles as prescribed by their physician.

Injection 2 occurred on Day 8 and was administered at the study clinic by the patient using the prefilled pen following training provided by the clinic site Trainer/Observer. During this injection, the Trainer/Observer observed patient use of the prefilled pen and reinforced training as needed. Injection 3 occurred on Day 15 and was self-administered by the patient at home using the prefilled pen. The final injection, Injection 4, occurred on Day 22 and was administered at the study clinic by the patient using the prefilled pen. At this clinic visit the Trainer/Observer observed patient use of the prefilled pen and completed a detailed questionnaire documenting patients' ability to self-inject with the device. The Trainer/Observer completed the observation in a hands off manner; no assistance or correction was provided to the patient during this final injection. In total, patients were to receive one injection with the prefilled syringe and three injections with the prefilled pen.

Evaluations were made at various time points throughout the study. The treatment schedule and corresponding evaluations are displayed in Table 1.

**Table 1:** Treatment period and evaluation schedule

	Screening visit		Injection using the prefilled syringe				Injections using the prefilled pen			EOS or EW
	**Clinic**	**Clinic**	**Clinic**	**Clinic**	**Clinic**	**Clinic**	**Clinic**	**Home**	**Clinic**	**Clinic**

	**14-day period**	**Day 1**	**Day 2**	**Day 3**	**Day 8**	**Day 9**	**Day 10**	**Day 15**	**Day 22**	**Day 23**

**Treatment and evaluations**		**Injection 1**			**Injection 2**			**Injection 3**	**Injection 4**	

Study enrollment		x								

Injection using the prefilled syringe		x								

Neopterin serum sample collection		x^a^	x^b^	x^c^	x^a^	x^b^	x^c^			

Prefilled pen training					x					

Injection using the prefilled pen					x			x	x	

Patient assessment of the prefilled pen training materials					x			x	x	

Patient assessment of injection site pain		x^d^			x^d^			x^d^	x^d^	

Patient assessment of ease of use		x			x			x	x	

Patient assessment of injection procedure		x			x			x	x	

Clinician assessment of injection site		x^e^	x		x^e^	x			x^e^	x

Observation form									x	

Preference questionnaire										x

Patient assessment and dosing information forms dispensed					x					

Concomitant therapy and adverse events	Monitor and record throughout study									

EOS, end of study; EW, early withdrawal.

^a^Neopterin samples were to be obtained 1 hour pre-injection.

^b^Neopterin serum samples were to be obtained 24 hours ± 2 hours post-injection.

^c^Neopterin serum samples were to be obtained 48 hours ± 2 hours post-injection.

^d^To be completed within 1 hour before injection and also immediately after injection.

^e^Clinician injection site assessment was to be performed within 1 hour before injection.

### Study Endpoints

#### Primary

The primary assessment of the safe and effective use of the prefilled pen was to evaluate the overall success rate as measured by the proportion of patients who successfully used the device. Data for determining the overall success rate were generated from an observation form that was completed by the Trainer/Observer during the final injection (Day 22). The observation form was composed of a series of questions organized around the three key steps of the injection process: device setup, self-administration of injection, and capping and disposal of the device. All actions captured in the observation form that would define the patient's handling of the prefilled pen as a failure were pre-defined in the protocol. Failure was further categorized as "failure patient induced" or "failure-possible device malfunction." Overall success was defined as no failures occurring during the patient's use of the device.

#### Additional

Additional endpoints in this study included patient assessments, clinician assessments, a pharmacodynamic evaluation, and safety monitoring.

##### Patient Preference Assessment

A preference questionnaire was administered to the patients on the last clinic visit (either Day 23 or the final visit if patient was withdrawing early from the study). The questionnaire investigated whether patients preferred the prefilled pen or the prefilled syringe, and scored patient preference for specific features of the prefilled pen compared to the prefilled syringe using a grading scale ranging from 0 (defined on the form as "much worse") to 10 (defined on the form as "much better"); definitions for integers 2-9 on the scale were not specified.

##### Patient Assessment of Injection Procedure

The injection procedure was assessed by each patient in order to evaluate whether patients experienced any difficulty with the processes of preparing, injecting, removing, and disposing after each of the 4 injections (Days 1, 8, 15, 22).

##### Patient Assessment of Injection Site Pain

Injection site pain was evaluated pre and post injection by patients for each of the 4 injections (Days 1, 8, 15, 22). Pain was evaluated on a scale from 0 (no pain) to 10 (extremely painful).

##### Patient Assessment of Ease of Use

Patients evaluated the "ease of use" for each of the 4 injections performed (Days 1, 8, 15, 22) by using the Ease-of-Use Grading Scale to indicate how easy it was for the patient to perform the injection. The scale ranged from 0 (extremely difficult) to 10 (extremely easy).

##### Patient Assessment of Training Materials

For each use of the prefilled pen (Days 8, 15, 22), patients evaluated how easy or difficult it was to read and understand the training materials.

##### Clinician Injection Site Assessment

Clinicians at the study site assessed injection sites 1 hour before injection and 24 hours following injection for each administration that took place at the clinic (Days 1, 8, 22). The injection site was examined for erythema, induration, temperature, and tenderness.

##### Pharmacodynamics

In order to evaluate levels of neopterin, a well-established biological marker of pharmacodynamic response to activation of the Type 1 interferon receptor, blood samples were collected from patients one hour prior to injection and 24 and 48 hours following injection for Injection 1 (Day 1, prefilled syringe) and Injection 2 (Day 8, prefilled pen).

##### Safety

Adverse events were monitored throughout the study.

### Statistical Analyses

A 10% dropout rate was assumed for this study. A sample size of 70 patients was required to provide a 95% confidence interval of the success rate [82.6%, 97.4%] based on the assumption that 90% of patients would successfully use the prefilled pen. The population used to evaluate the primary outcome consisted of those patients who received Injection 1 with the prefilled syringe, received at least one injection with the prefilled pen, and had a completed observation form.

Summary and descriptive statistics were used in this study. No formal statistical testing was preplanned in the protocol.

## Results

### Patient demographics

Of the 74 enrolled patients, 64 (86%) were female. Mean patient age was 49.6 years (range: 22 to 65 years). The mean body mass index (BMI) of the study population was 28.92 kg/m^2 ^(range: 17.9 to 42.2 kg/m^2^).

### Patient Exposure to Study Treatment

Of 74 enrolled patients, 71 patients (96%) completed the study. All 74 enrolled patients received a self-injection using the prefilled syringe. Two patients discontinued from the study following the injection with the prefilled syringe prior to receiving an injection with the prefilled pen (one patient was withdrawn after missing two clinic visits and the other patient was withdrawn due to a MS relapse). Seventy two patients received at least one self- injection with the prefilled pen and 71 patients completed all three injections with the device. One patient completed the final injection but did not have a completed observation form. As a result, the observation form was only completed for 70 patients. In total, 215 injections were administered with the prefilled pen.

### Overall Success Rate

The overall injection success rate was 89% (62/70 patients). No failures due to device malfunction and no damaged or bent needles were reported. Eight (11%) patient induced failures occurred, the majority of which took place during device setup (seven patients removed the needle cap manually rather than by extending the injector shield). None of these events resulted in patient injury and all patients were able to complete administration with the prefilled pen. Patient-induced failures resulting in the device becoming unusable occurred in one patient. In this case the patient did not follow instructions and removed the device from the thigh prematurely before medication was administered; upon a second attempt the patient was able to successfully complete all steps of the injection process. Patient success at each observation of self-administration using the prefilled pen is described in Table 2.

**Table 2:** Patient success at each self-administration step using the prefilled pen (analysis population, n = 70)

Steps in self-administration using the prefilled pen	Patients who completed step correctly and in an optional manner, *n *(%)
**Device setup**	
Holds device in an upright position and removes tamper-evident cap	66 (94)
Holds device in an upright position and attaches needle	58 (83)
Extends injector shield all the way, while pointing device away from body	61 (87)
**Injection**	
Places and holds prefilled pen perpendicularly to anterior lateral thigh (injection site)	68 (97)
Applies firm downward pressure on the body of the prefilled pen and releases the safety lock and fires device by depressing blue activation button	65 (93)
Holds device for a count of 10 seconds before removing needle from thigh	67 (96)
Lifts device straight out, perpendicular to thigh	68 (97)
Visually confirms delivery via circular display window	58 (83)
**Capping and disposal**	
Caps the device with blue cover	66 (94)
Does not hold blue cover in place while capping prefilled pen	63 (90)

Note: All data were captured in the observation form.

### Patient Preference Assessment

**Figure 2:**
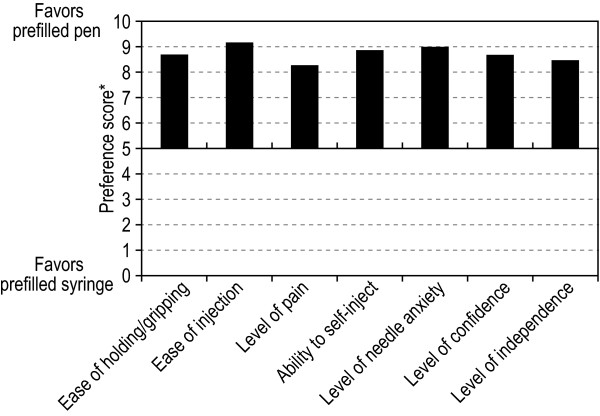
**Mean patient preference scores for prefilled pen vs. the prefilled syringe on 7 domains relevant to self-injection (analysis population, n = 70)**. *Scores in each domain range from 0 (prefilled pen much worse) to 10 (prefilled pen much better).

**Figure 3:**
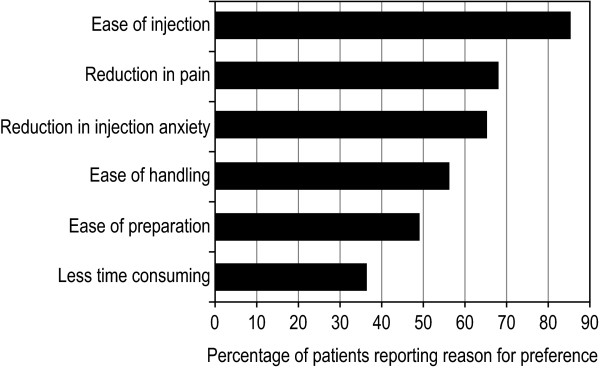
**Most common reasons reported for preferring the prefilled pen (analysis population, n = 70)**.

The majority (94%) of patients indicated a preference for the prefilled pen over the prefilled syringe. Patients evaluated the prefilled pen in comparison to the prefilled syringe using a grading scale ranging from 0 (much worse) to 10 (much better). Across all domains, patient preference for the prefilled pen was strong (Figure 2). Patient preference was related to key features of the injection process, including ease of holding and gripping (mean score of 8.7), ease of injection (mean score of 9.2), level of pain (mean score of 8.3), level of independence (mean score of 8.5), level of confidence (mean score of 8.7), and needle anxiety (mean score of 9.0). The most common reasons for patient preference for the prefilled pen are listed in Figure 3.

### Patient Assessment of Injection Site Pain

Following injection with the prefilled syringe, the mean pain score was low, 1.7 (out of 10). The mean pain score for each of the 3 injections with the prefilled pen was also low: 1.0, 1.3, and 0.7 for injections on Days 8, 15, and 22, respectively.

### Patient Assessment of Injection Procedure

Eighty-nine percent of patients reported having no difficulty with administration using the prefilled syringe. For each of the injections using the prefilled pen, 90% of patients reported having no difficulty.

### Patient Assessment of Ease of Use

The mean ease of use score for the injection administered with the prefilled syringe was 8.1 (out of 10). For each administration with the prefilled pen, the mean ease of use score was as follows: 8.9 on Day 8, 8.7 on Day 15, and 9.3 for the final injection with the prefilled pen.

### Patient Assessment of Training Materials

Patients referred to the written instructions and DVD less with each subsequent injection using the prefilled pen. Of the patients who used the written instructions and DVD, the majority rated them as "very effective" in educating on how to use the prefilled pen (90%-93% for written instructions; 88%-90% for DVD instructions).

### Clinician Injection Site Assessment

Clinical injection site assessments made before and after injection were similar between the prefilled syringe and the prefilled pen. For the majority of patients, the clinician found no presence of induration, no temperature variation, and no tenderness at the site following injection with the prefilled syringe or the prefilled pen. Mild erythema was reported in 26% of patients after using the prefilled syringe and in 25% and 23% of patients on Days 8 and 22 after using the prefilled pen. Mild induration and mild tenderness were reported in less than 8% of patients following injections with both the prefilled syringe and the prefilled pen. There were no severe reports of erythema, induration, tenderness and temperature following any injection.

### Pharmacodynamics

Similar increases in mean neopterin serum levels were observed over time following administration with the prefilled pen (6.2 ng/ml before injection, 12.6 ng/mL 24 hours after injection, and 13.7 ng/mL 48 hours after injection) as were seen with the injection using the prefilled syringe (5.6 ng/mL before injection, 10.0 ng/mL 24 hours after injection, and 11.0 ng/mL 48 hours after injection). The mean neopterin induction ratios were similar for injection with the prefilled syringe and the prefilled pen (2.149 and 2.514, respectively).

### Safety - Adverse Events

All patients who received at least one dose of IM IFNβ-1a using either the prefilled syringe or the prefilled pen were included in the safety population. The overall incidence of AEs in this study was low and rates were similar between the prefilled syringe and the prefilled pen. One patient experienced a MS relapse during the study period. Eleven percent of patients reported an adverse event with use of the prefilled syringe (Day 1), and 17%, 18%, and 4% of patients reported an adverse event with each injection using the prefilled pen (Days 8, 15, and 22). No safety concerns potentially associated with the prefilled pen were observed. The incidence of injection site reactions was low and pain was infrequently reported with the prefilled pen (Table 3).

**Table 3:** The most common (≥3%) treatment-emergent prefilled pen injection site-related adverse events

	***n***	%
Number of patients who received at least 1 injection with prefilled pen	72	100

**Injection site-related adverse event**		

Injection site pain	5	7

Injection site hematoma	4	6

Injection site erythema	2	3

Injection site hemorrhage	2	3

Injection site induration	2	3

Note: A patient was counted only once within each preferred adverse event term.

## Discussion

This open-label study evaluated the safe and effective use of the prefilled pen in patients currently self-administering IM IFNβ-1a via the prefilled syringe. These patients represent the population expected to use the prefilled pen and as such, were believed to be well suited to evaluate the prefilled pen.

Safe and effective use of the prefilled pen was assessed from data captured in the observation form completed by the Trainer/Observer. The comprehensive list of questions in the observation form was developed to provide an overall evaluation of the patients' ability to properly self-inject with the device during the final injection using the prefilled pen. There were no device malfunctions and the overall success rate of the prefilled pen was high (89%), demonstrating that it provides a safe and effective alternative method of administering IM IFNβ-1a. Patients also gave high ratings to the related training materials and injection procedure.

The prefilled pen was specifically designed to overcome multiple challenges of self-injection faced by patients with MS. Features designed to reduce injection anxiety include a protective shield that conceals the needle and automated needle insertion and medication dispensing, which reduces the number of steps involved in the dosing process. Safety features include a mechanism to prevent early injection as well as a visual indicator that allows for confirmation of injection process completion. The diameter and length dimensions are designed to help stabilize the device during the injection process so as to improve ease of use for patients with impaired motor coordination. In this study, 94% of patients preferred the prefilled pen over the prefilled syringe. Reasons for patient preference for the prefilled pen were related to ease of holding and gripping, ease of injection, level of pain, and needle anxiety, confirming that the design of the prefilled pen was successful in making the injection process easier. In addition, although the study was not originally designed to compare the prefilled syringe to the prefilled pen, a post hoc paired *t *test was performed to compare the ease of use assessment at Injection 1 (Day 1, with the prefilled syringe) with Injection 4 (Day 22, with the prefilled pen). Results demonstrated that patients found the prefilled pen statistically significantly easier to use after 3 injections compared to the prefilled syringe after at least 12 uses, as required by study entry criteria (mean ease of use scores 8.1 and 9.3, respectively).

Automated injection devices offer a means to potentially reduce injection site pain. In this study, patient assessment of pain was low for the injection with the prefilled syringe (1.7 out of 10), and numerically lower for each of the three injections with the prefilled pen. A post hoc comparison of the pain assessment at Injection 1 (Day 1, with the prefilled syringe) and Injection 4 (Day 22, with the prefilled pen) was performed by paired *t *test and showed that patients experienced statistically significantly less pain with the prefilled pen by the third use than with the prefilled syringe after at least 12 uses, as required by study entry criteria (mean pain scores 1.7 and 0.7, respectively). The incidence of pain through safety monitoring was also low for both methods of administration. Seven percent of patients reported an AE of pain related to use of the prefilled pen and none of the reports were severe. In addition to pain assessments, the injection site was assessed at multiple time points during the study by the clinician. Mild erythema was observed at a similar frequency following injection with the prefilled syringe and injection with the prefilled pen. The incidence of other injection site reactions such as temperature, induration, and tenderness were infrequent, and rates were similar for the two injection methods.

Importantly, there were no new safety concerns raised in this study. The safety profile observed with the prefilled pen was similar to the known safety profile of IM IFNβ-1a from the prescribing information and post marketing data.

Although IM injections are frequently administered via a manual syringe with a longer needle, the 25 G × 5/8 inch (16 mm) needle was determined to be the appropriate size for use with the prefilled pen. Earlier development of the prefilled pen using a 23 G × 1.25 inch (30 mm) needle indicated that this length was not appropriate for use with the device due to needle bends, which did not occur in this study with use of the shorter needle. The ability of the needle used in this study to deliver an IM injection is supported by published reports indicating that a 5/8 inch needle (16 mm) can be expected to access the IM space in the majority of patients when applied with a manual syringe [21-23]. Considering the compressive effects related to the forceful application of the prefilled pen to the cutaneous tissue, the shorter needle is likely appropriate for general use [22,24].

As discussed earlier, reduction in needle anxiety and injection pain were amongst the subjective benefits specifically sought in the design of the prefilled pen. We note that the shorter needle length of the prefilled pen may, in addition to its other features, contribute to the observed patient preferences for this device over the prefilled syringe. Our observation that neopterin induction was similar following injection with the prefilled syringe and with the prefilled pen further supports that the 25 G × 5/8 inch (16 mm) needle is the appropriate size to effectively deliver the full dose of IM IFNβ-1a with the prefilled pen as designed.

There are limitations to consider when interpreting the results of this study. First, we acknowledge that the questionnaires and assessments used in the study have not been formally validated. However, they were specifically developed to capture information relevant to the robust evaluation of the safety and efficacy of the prefilled pen.

In addition, several features of the study design and population should be emphasized. We enrolled only patients actively interested in the prefilled pen. In this regard, we are not able to speculate on the outcomes of a similar study conducted in patients who were not interested in a self-injection device. Given the nature of the study, it was also impossible to blind patients and study staff regarding injection method. As such, the data derived from the subjective assessments made in this study may partially reflect patients' and clinicians' expectations for the prefilled pen. The partial crossover study design in which patients switched from injections with the prefilled syringe to injections with the prefilled pen (and not from the prefilled pen to the prefilled syringe) does not allow us to exclude an impact of treatment order on our findings. Additionally, patients evaluated the prefilled syringe injection experience only once, whereas they evaluated the prefilled pen injection experience a total of three times. It could be argued that patient responses may have varied when assessing the different methods since there were repeat evaluations for only one of the methods. However, since the patients in the population selected for this study were currently using the prefilled syringe, it is unlikely that the single assessment of the prefilled syringe would have differed significantly if that assessment had been repeated.

While we acknowledge that this study evaluated the prefilled pen in patients currently self-administering IM IFNβ-1a via the prefilled syringe and therefore does not address its suitability in treatment-naïve patients or when utilized by caregivers, the patient preference and ease of use results indicate the prefilled pen would also be an attractive option for use in these populations.

## Conclusions

Injection anxiety and physical limitations are major barriers to self-injection for many patients with MS. These barriers may contribute to poor treatment adherence that may result in a reduction in the clinical benefits of MS therapy.

Results from this study support the safe and effective use of the prefilled pen for self-administration of IM IFNβ-1a by patients with MS. Patients preferred the prefilled pen over the prefilled syringe for reasons related to ease of use, injection pain, needle anxiety, and sense of independence. Data from this study demonstrate the potential for the prefilled pen to fulfil an unmet need in patients using IM IFNβ-1a by offering an alternative method for IM delivery that simplifies the injection process. The prefilled pen provides patients the opportunity to gain the clinical benefits of IM IFNβ-1a treatment while improving their ability to independently manage their disease.

## Competing interests

**JTP **has participated in consulting and/or speakers' bureau with Biogen Idec, Genzyme, Novartis, and Teva Neuroscience, and has participated in clinical research with Biogen Idec.

**EF **has participated in consulting and/or speakers' bureau with Bayer, Biogen Idec, EMD Serono, Genzyme, Opexa, Novartis, Pfizer, and Teva Neuroscience, and has participated in research studies involving Biogen Idec, Eli Lilly, EMD Serono, Genzyme, Ono, Roche, Sanofi Aventis, and Teva Neuroscience.

**WG **has participated in consulting, speakers' bureaus, and clinical research with Biogen Idec and Teva Neuroscience.

**DT, SL**, and **AD **are employees of Biogen Idec.

## Authors' contributions

All authors contributed to the manuscript and have read and approved the final version. JTP served as the principal investigator and participated in the study design. JTP, EF, and WG served as study site investigators. DT participated in the conduct of the study and data analysis. SL participated in the study design and performed the statistical analysis. AD participated in the data analysis.

## Pre-publication history

The pre-publication history for this paper can be accessed here:

http://www.biomedcentral.com/1471-2377/11/126/prepub
